# Clinical outcome prediction in pediatric respiratory infections using hybrid feature selection and a genetic algorithm-optimized machine learning

**DOI:** 10.1038/s41598-025-18258-6

**Published:** 2025-10-21

**Authors:** Sarlinraj Madhalaimuthu, Sujatha Radhakrishnan

**Affiliations:** School of Computer Science Engineering and Information Systems, Vellore Institute of Technology, Vellore, Tamilnadu 632014 India

**Keywords:** Ensemble machine learning, Pediatrics, Clinical outcomes prediction, BD IRA dataset, Health care, Medical research

## Abstract

Respiratory ailments constitute various pathological conditions affecting the respiratory system, including the airways, pulmonary tissues, and associated structures. When these conditions are left untreated or inadequately managed, they can result in long-term complications, diminished life quality, and higher death rates. To alleviate the strain of respiratory illnesses and promote a more robust population, it is crucial to focus on raising public awareness, facilitating early detection, implementing preventive strategies like immunization, and furthering medical advancements in treatment options. The study presents a comprehensive Machine Learning (ML) method to improve the investigation and classification of respiratory datasets. The technique applies data preprocessing, augmentation, feature selection, genetic algorithms, and ensemble learning techniques on a “Respiratory dataset” and achieves high predicted accuracy while maintaining interpretability. The Synthetic Minority Oversampling Technique (SMOTE) is used to address data imbalance and ensure proper representation of minority class samples. The feature selection module uses various strategies to find relevant characteristics and reduce dimensionality. Machine learning algorithms that are apt for the dataset are employed for predicting the target variable; their performance is measured and analyzed thoroughly. By using Genetic algorithms, Random Forest, XGBoost, and Gradient Boosting are selected as optimal models. The ensemble learning framework combines the 3 optimal models and creates a strong classification system to predict “target variable : Clinical Progression” output. The performance measures of the proposed model achieved an overall accuracy of 95.02% when compared with the existing works and can be applied in healthcare analytics.

**Supplementary Information:**

The online version contains supplementary material available at 10.1038/s41598-025-18258-6.

## Introduction

Global health is at risk due to respiratory disorders, which require extremely reliable, efficient, and effective diagnostic methods. Respiratory diseases have far-reaching consequences that go beyond personal health, impacting entire families, communities, and healthcare infrastructures. Lung diseases, both infectious and non-infectious, are the most prevalent cause of mortality overall in the world Kumar et al.^1^. The global disorders have exhibited a marked upward trend, attributable to various factors such as environmental pollution, shifts in lifestyle patterns, and heightened exposure to both allergenic substances and pathogenic microorganisms. Untreated or poorly managed conditions can lead to chronic complications, reduced quality of life, and increased mortality rates. Promoting awareness, early diagnosis, preventive measures such as vaccination, and advancements in medical treatment are critical steps toward mitigating the burden of respiratory diseases and fostering a healthier population. Asthma, pneumonia, and chronic pulmonary disease are the most common respiratory conditions globally. Effectively addressing respiratory disorders necessitates early detection, accurate diagnosis, and proper management. Recent advancements in data-driven technologies like data augmentation, feature selection, genetic algorithm, ensembling methods, machine learning (ML) models…etc. are integrated to improve the efficiency of the prediction. Data augmentation is crucial for improving model performance especially when dealing with sparse or unbalanced datasets. By creating artificial variations of existing data, such as augmented chest X-rays or transformed patient information, it helps machine learning models generalize better to previously unseen scenarios. Feature selection can be used to identify the most relevant characteristics for predicting the output. This process enhances model accuracy by eliminating unnecessary or irrelevant features, reduces computational complexity, and helps prevent overfitting. Ultimately, this results in more reliable and interpretable models Hussain et al.^2^.

Research on respiratory diseases uses a variety of machine learning models, such as Supervised Learning Models—for both regression and classification tasks, such as predicting lung function or identifying various types of illnesses, Unsupervised Learning Models—to identify hidden patterns within data or to categorize patients into different risk groups, Deep Learning—for image-based diagnostics, such as identifying respiratory disorders by examining X-rays or CT scans. ML methods can identify patterns in input data and helpful to make informed predictions. These models are commonly used to address respiratory issues, including diagnosing conditions, predicting disease progression, and optimizing treatments. As we use feature selection, which may affect the performance of the ML model, Genetic Algorithm (GA) is utilized to select the optimal model and achieve better accuracy in the prediction Maleki et al.^3^. As serve as optimization techniques for natural selection and evolution. These algorithms are commonly applied in respiratory data for tasks such as feature selection, hyperparameter tuning, and enhancing diagnostic or predictive models. By mimicking biological evolution, GAs proficiently navigate extensive search spaces to pinpoint optimal solutions for complex problems.

Combining various ML models enhances the robustness and accuracy of predictions. The Ensemble method is used to merge different ML models’ predictions and intensify the performance of the final model with optimized performance Ali et al.^4^. Strategies like bagging, boosting, and stacking leverage the strengths of each model while reducing its weaknesses. For example, ensemble methods, including random forests and gradient boosting machines, are commonly used to amalgamate predictions from multiple classifiers, thereby improving the accuracy of respiratory disease detection. Incorporating advanced machine learning methods, such as data augmentation, feature selection, genetic algorithms, and ensemble techniques, can significantly transform how respiratory issues are tackled. These strategies boost diagnostic accuracy, enhance predictive power, and aid in creating personalized treatments, leading to improved patient outcomes and more effective healthcare systems.

In summary, this research proposes a hybrid machine learning framework tailored for pediatric respiratory outcome prediction, integrating rigorous data preprocessing, ten diverse feature selection techniques, and a genetic algorithm for optimized model selection. A novel fitness function, incorporating eight clinical performance metrics, is employed to identify the top three models, which are then combined using a soft voting ensemble approach. The proposed method demonstrates superior predictive accuracy, robustness, and clinical relevance when validated on the BD IRA dataset. To our knowledge, this is one of the first works to employ such a comprehensive pipeline combining multi-method feature selection and evolutionary model selection for outcome classification in pediatric respiratory infections.

**Fig. 1:**
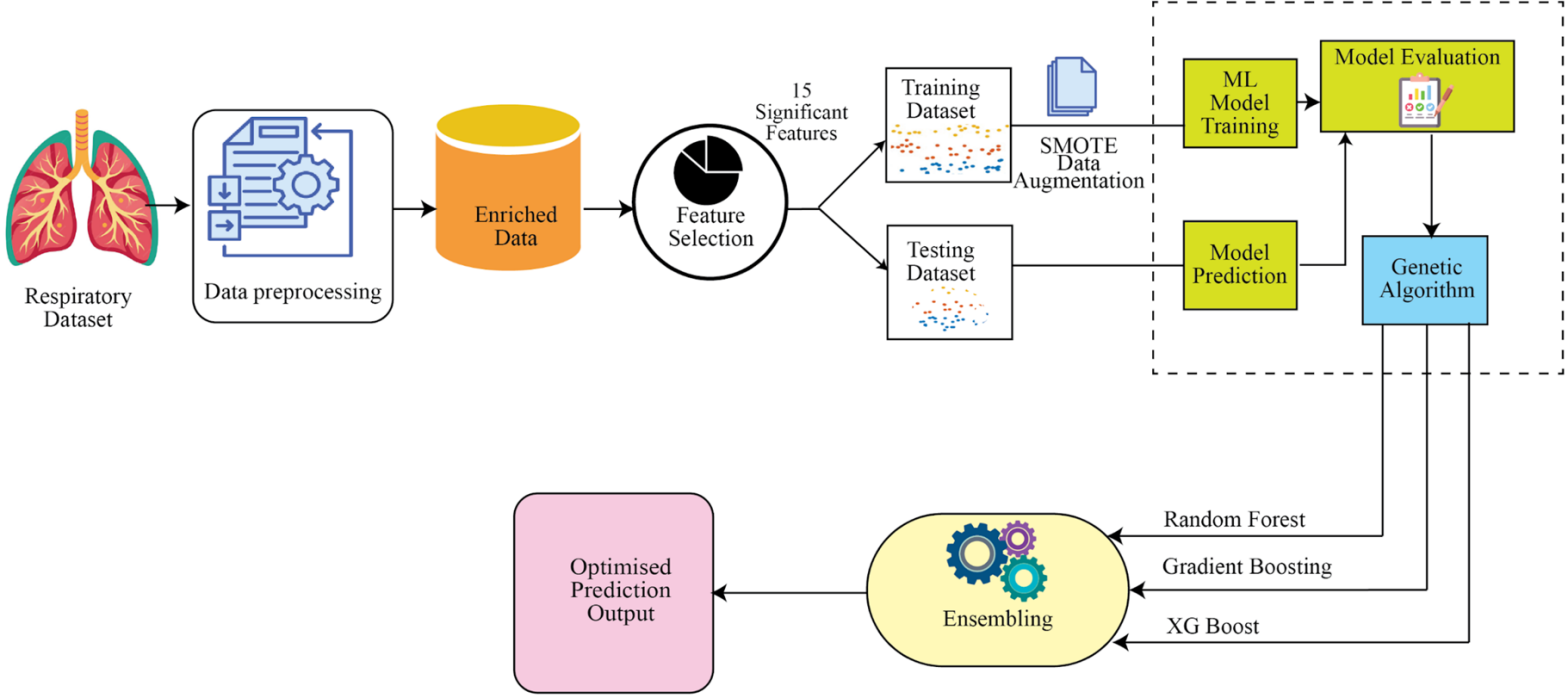
Architecture of the proposed model.

This is how the rest of the paper is organized. In Section"Review of literature", the suggested work’s pertinent research background is explained. Section"Problem statement"clearly defines the problem statement and the dataset of the work. Section"Proposed methodology"provides a detailed explanation of the comprehensive research. Furthermore, a brief graphical abstract of the proposed method is given in Fig. 1. A clear step-by-step explanation of the proposed approach and outcomes is demonstrated in Section"Proposed methodology". This research is specifically useful in the healthcare domain for predicting “target variable: Clinical progression” choices based on the respiratory dataset. Lastly, Section"Conclusion"provides the conclusion.

## Review of literature

Before beginning the research, a comprehensive Literature review was conducted in four directions, examining 54 articles from various research databases, digital libraries, and search engines such as SCOPUS, Web of Science, Springer, IEEE, and others, to establish a solid foundation of knowledge and identify any unmet, further research needs in this field.

### Direction A—feature selection methods

In the research of Sultan & Saadeh^5^ to determine important features from photoplethysmogram signals for estimating respiratory rate and blood pressure, the experts probed a correlation-based feature selection approach and achieved accurate estimations. A comparative study of various feature selection algorithms for the categorization of respiratory disorders was carried out by Gürkan Kuntalp et al.^6^ IG, which emphasized the significance of efficient feature selection in raising classification accuracy.

In Emam et al.^7^, several feature selection methods were used with medical data and found pertinent features, to improve the effectiveness of ML models in medical diagnosis. It was not able to capture the interactions between features, which is mandatory for medical data. Xia et al.^8^ developed SVM-RFE (Support Vector Machine Recursive Feature Elimination) to divide COPD patients according to how well they follow treatment instructions and achieved an AUC of 0.987. Tarek et al.^9^ offered a feature selection strategy based on Snake Optimization Algorithm so that the cardiovascular diseases can be detected quickly. This approach is bio-inspired and had a high classification rate which reduced the dimensionality of the feature and thus demonstrated the possible significance of evolutionary algorithms in the formulation of quality healthcare solution which is interpretable.

An improved version of the traditional SVM-RFE algorithm was proposed in D. Zhang et al.^10^ to analyze the respiratory data by adding a correlation bias reduction technique and distinguishing healthy and diabetic individuals Y.-H. Hu et al.^11^. Suggested a novel way to deal with missing values in medical datasets by combining RFE with imputation method. It enhanced imputation quality for better performance but overfitted the training data. 

Gürkan Kuntalp et al.^6^ had used metaheuristic feature selection methods to classify respiratory diseases. These methods effectively reduced data dimensionality and enhanced the accuracy, but instability, overfitting, and bias toward dominant features are some of the challenges in this research. In the study of Haque et al.^12^ five publicly benchmarked datasets were used to test their algorithms, proving the effectiveness of the SA-based feature selection technique but faced problems like parameter sensitivity, computational intensity, and SA’s time consumption. Inbarani et al.^13^ Presented a supervised feature selection technique that combines an enhanced Harmony Search (HS) algorithm with Rough Set Quick Reduction to manage complicated medical data by successfully reducing dimensionality and improving the precision of classification in medical data.

A system for segmenting and analyzing lung CT scan images for COVID-19 identification was created by Rajinikanth et al.^14^ using Harmony Search (HS) optimization and Otsu thresholding for respiratory disease diagnosis. An adaptable deep feature selection approach was proposed by Pramanik et al.^15^ for the identification of pneumonia by combining PSO with deep learning features taken from pre-trained models. A new RegNet and XOR-based PSO method for choosing deep features in pneumonia diagnosis was presented by Ladani & Semnani^16^ that produced high levels of accuracy. For high-dimensional medical datasets, Sekhar & Vijayakumar^17^ suggested a feature selection technique that combines ACO with a weighted visibility graph that improves the performance of classification.

### Direction B: multiple machine learning models for respiratory issues

The study by Molfino et al.^18^ used Logistic Regression (LR) to forecast asthma flare-ups by examining several patient characteristics and achieved an AUC of 0.785 J. Hu et al.^19^. introduced the acute respiratory distress syndrome (ARDS) Mortality Prediction Model, which uses Logistic Regression (LR) to predict mortality risk in ARDS patients but this approach is not well suited for complicated datasets. Dual-Tree Complex Wavelet Transform (DTCWT) with Linear Discriminant Analysis (LDA) were integrated in Aujla et al.^20^ to categorize lung ultrasound pictures. In Wang & Safo^21^, LDA was used to find molecular signatures that distinguish across patient groups with different levels of disease severity. However, LDA is more sensitive to outliers and has scalability problems.

Xia et al.^8^ Created a model utilizing Support Vector Machine (SVM) in conjunction with Recursive Feature Elimination (SVM-RFE) to categorize COPD patients with an AUC of 0.987. Cough detection model was proposed in Chen et al.^22^ using SVMs to categorize cough episodes from audio inputs and predicted 94.9% accurately. Yoo et al.^23^ Created a deep learning-based decision tree classifier for identifying COVID-19 in chest X-ray pictures with 91% accuracy. The decision tree model was used by Yang et al.^24^ to estimate mortality in patients (AUC = 0.85) with severe COVID-19 Khalilia et al^25^. Successfully handled unbalanced data by using Random Forest (RF) to forecast disease risk. A RF-based monitoring system was created in Alam^26^ to forecast patient mortality and length of stay in ICUs. In Li et al.^27^ PRF-RW, a progressive RF-based approach is introduced to diagnose respiratory risks by using semi-automated segmentation of pulmonary lobes, but this method faced some challenges, like computational complexity.

Tripathi^28^ used the Gradient Boosting Model (GBM) to predict the outcomes of SARS-CoV-2 infections. An Extreme Gradient Boosting (XGBoost) model was proposed in Jha et al.^29^ to identify pulmonary fibrosis in COVID-recovered individuals. Nishio et al.^30^ estimated respiratory rates from photoplethysmogram signals by combining Gradient Boosting technique with power spectral feature extraction. In, Bayesian optimization and Gradient Boosting were used to assess a computer-aided diagnosis system for lung-tumor categorization. Lyu & Nakayama^31^ Deployed ensemble algorithms to predict respiratory failure in ICU patients Zhao et al.^32^. Proposed a BiGRU-Attention-XGBoost model to classify respiratory sounds for early detection of respiratory risks.

According to El-Kenawy et al.^33^, Greylag Goose Optimization (GGO) algorithm implementation and Multilayer Perceptron (MLP) are recommended to perform lung cancer classification. It was found to be effective in collaboration with the diagnosis method by a synergy of bio-inspired optimization and neural net weight adjustment GGO medium/high in clinical field. Sreerama^34^ used multi-layer perceptrons (MLPs) to classify the healthy-unhealthy respiratory noises. The classification of pulmonary function using MLP neural networks was examined in Almazloum et al.^35^ with precision varied between 87 to 92% Zhu et al.^36^ Utilized MLPs to integrate deep learning and radiomics characteristics to create a preliminary diagnostic model for COPD.

Prince et al.^37^ proposed a technique using LBP, YCrCb color space, and CLAHE for feature extraction from chest X-rays Al-Aidaroo et al.^38^. compared Naïve Bayes with five classifiers on 15 medical datasets and proved that Naive Bayes was better for medical applications. Sachdeva et al.^39^ Suggested a unique method “Pearson Correlation Weighted K-Nearest Neighbor (PCWKNN)” for the diagnosis of lung cancer Palaniappan et al.^40^ evaluated k-NN with Support Vector Machine (SVM) classifiers to diagnose respiratory diseases using pulmonary acoustic data.

### Direction C: feature selection methods and machine learning models in medical data

In recent research, Huang et al.^41^ developed an ensemble learning-based feature selection method that improves performance and accuracy, particularly for time-series prediction. A hierarchical ensemble technique is proposed in Tumay et al.^42^ for an in-depth feature selection Alzakari et al.^43^ Also suggested an improved LSTM-based model that can accurately predict agricultural prices compared to traditional models in terms of forecasting accuracy over time. Although the field is agro-economic, the approach is also applicable in a healthcare setting, when studying the progress of a disease or monitoring patient outcomes.

In Begum et al.^44^, the Weighted Rank Difference Ensemble (WRD-Ensemble) is a feature selection method that combines Pearson’s correlation coefficient, ReliefF, and Gain Ratio Saeys et al.^45^ Used ensemble feature selection techniques to improve the reliability in multidimensional datasets with minimum samples. UBayFS (User-guided Bayesian Framework for Ensemble Feature Selection in Life) was proposed by Jenul et al.^46^ in 2022 to produce flawless and reliable feature selection for respiratory research. To forecast ARDS (Acute Respiratory Distress Syndrome) after heart surgery, H. Zhang et al.^47^ investigated tree-based ensemble ML models. Espinosa et al.^48^ utilized a multi-objective ensemble learning strategy for time-series forecasting in Long Short-Term Memory (LSTM) networks.

### Direction D: feature selection methods, machine learning models and genetic algorithms in medical/respiratory data

To forecast respiratory disorders, Kaur et al.^49^ used an ensemble model by combining feature selection using binary grey wolf optimization with hyperparameter optimization using genetic algorithms and achieved high predictive accuracy. To improve the stability and applicability of selected attributes, Battistella et al.^50^ developed Graphical Ensembling (GE), an ensemble feature selection technique based on graph theory. Pasha & Mohamed^51^. Presented a Bio-inspired Ensemble Feature Selection (BEFS) model in conjunction with machine learning techniques for illness risk prediction.

After a detailed literature study, various feature selection techniques like Filter, Wrapper, Embedded, Physics-Inspired, and Nature-Inspired methods are used, ML models, Genetic algorithms, and ensemble techniques are applied in the current research for optimal and ideal output.

## Problem statement

Even though the works that are considered in the literature review have numerous traditional and modern approaches, most of them have shortcomings, such as computational complexity, sensitivity to changes in parameters, unsaturated problems, time-consuming overfitting, unsteadiness, and so on. The research aims to tackle the concerns by the following steps:

- Conventional feature selection methods are used for selecting significant features.
- Suitable machine learning models are built, and their performance is evaluated by using metrics.
- Using genetic algorithms, three optimal models are chosen.
- The ensemble approach integrates the optimal three chosen models to build an effective model for predicting the outcome. The resulting, integrated model performance surpasses the current models and is outstanding.

The dataset used in this study is publicly available and can be accessed at: https://data.mendeley.com/datasets/2d9dvnycjw/1.

We renamed the above dataset as “BD IRA.xlsx” in our research for easy reference. It is a dataset on respiratory infections used in research to predict the “target variable: Clinical progression” which is a categorical feature type. There are three different classes in this “target variable: Clinical progression”. Class 0 is “Cured and discharged home”, Class 1 is “Death” and Class 2 is “Left against medical advice”.

Optimal Prediction of the “target variable: Clinical progression”—Class” by using the other features of the respiratory dataset is the motto of this research.

## Proposed methodology

To enhance the better performance measures, while maintaining resilience and interpretability, the proposed method combines data preprocessing, feature selection, model training, genetic algorithms, and ensembling to build an optimal model that will be more efficient and effective than the existing models.

Fig. 1 below provides a concise, step-by-step graphical representation of the complete architecture of the proposed model for predicting clinical outcomes in pediatric respiratory infections.

The initial step in the proposed workflow is data preprocessing, which will involve the renaming of the columns, missing values, encoding of categorical variables and dropping of unnecessary variables. After that, ten feature selection techniques spanning five categories including filter, wrapper, embedded, physics-based and nature-based are applied to sift out the most appropriate clinical features. With frequency voting, 15 most important features are chosen to develop the model. To overcome the possibility of difference between classes within the “target variable: clinical progression”, SMOTE technique is used on the training data only.

Then, 10 machine learning algorithms will be trained and tested on the eight performance measures. Then, a Genetic Algorithm (GA) is applied to choose the three most suitable models according to the weighted multi-metric fitness function. The chosen models—Random Forest, XGBoost, and Gradient Boosting will be combined by a smoothed voting ensemble, where the probability of classes is averaged to obtain a final prediction. The prediction is the category of “target variable: Clinical progression”: cured (0), dead (1) or left against medical advice (2).

### About the dataset

A comprehensive, step-by-step multimodal is developed in the research to improve the prediction of the outcome variable “target variable: Clinical progression” in the respiratory dataset “BD IRA.xlsx”. The respiratory dataset named “BD IRA.xlsx” with 91 entities and 801 records is used in this research Chawla et al.^52^. The dataset consists of clinical, biochemical, and epidemiological data for 801 children admitted to Rabat, Morocco, with respiratory infections. All clinical symptoms, laboratory test results, molecular diagnostic findings, and patient demographics are recorded in the dataset. “target variable: Clinical progression” is the output variable that will be predicted by the proposed model of this research.

### Data preprocessing

The data preparation processes aim to enrich the data for further study and model training through purification, modification, and preparation. It ensures the quality of the data and enhances model performance and derivation of reliable insights. Initially, the respiratory data obtained is preprocessed and cleaned. Removing the missing values, unnecessary data, naming conventions, and encoding is done on the dataset at the time of data preprocessing to standardize the data before proceeding with the research further. The pre-processing of the dataset is carried out as follows:

- Rename column names: Remove unnecessary spaces to ensure clarity and consistency in column references.
- Handling missing values
  - Identify the columns that are with > 5%missing values and drop them to avoid analysis errors.
  - Impute remaining missing values (< 5%) using the column mean to maintain dataset integrity.
- Data Transformation: Apply label encoding to convert categorical variables into numerical form for machine learning.
- Drop unnecessary columns: Remove the irrelevant columns like “ID PATIENT” and “Admission date” to prevent noise and bias in model training.

### Feature selection

In general, the feature selection techniques are leveraged to find the most pertinent characteristics and simultaneously reduce the dimensionality of the dataset. In our research, we have used 10 feature selection techniques that are listed out in the following Table 1.

**Table 1:** Feature selection methods used in the work.

S.No	Name of the feature selection method
1	Filter methods Correlation-based: Identifies features that are highly impacting the output Information gain: Addresses how each feature binds to the target variable and targets the significant elements
2	Wrapper methods Recursive feature elimination: Iteratively removes the least significant features based on model coefficients or importance scores Forward selection: Sequentially adds the best-performing feature to maximize model performance
3	Embedded methods L1 Regularization (Lasso): Penalizes less significant feature coefficients, shrinking them to zero to perform feature selection Decision tree feature importance: Measures the importance of a feature by evaluating how much it decreases impurity at decision nodes
4	Physics-inspired methods Simulated annealing: Optimizes feature selection by exploring the solution space and accepting probabilistic improvements Harmony search: Selects optimal features using an optimization algorithm inspired by musical harmony
5	Nature-inspired methods Particle swarm optimization: Optimizes feature selection by simulating particles (solutions) moving in search space Ant colony optimization: Uses a nature-inspired approach to find the optimal subset of features based on pheromone updates

The Rule of Thumb (10:1) in ML for feature selection states that the selected records should be at least 10 times the number of features selected. The 10 distinct feature selectors choose the most significant 20 features by following the common rule of thumb (10:1) and also by considering the nature of the data, the interdependency between the data, and other factors.

As our dataset consists of 801 records and 91 features, it is safer to consider approximately 90 features by following the rule, i.e., 10:1. However, these 90 numbers are required when we assume all the features are useful. In real-time analytics, because of other factors Like irrelevance, repetition, etc., the standard number of features that can be considered would be 10 to 30 after the feature selection process.

**Fig. 2:**
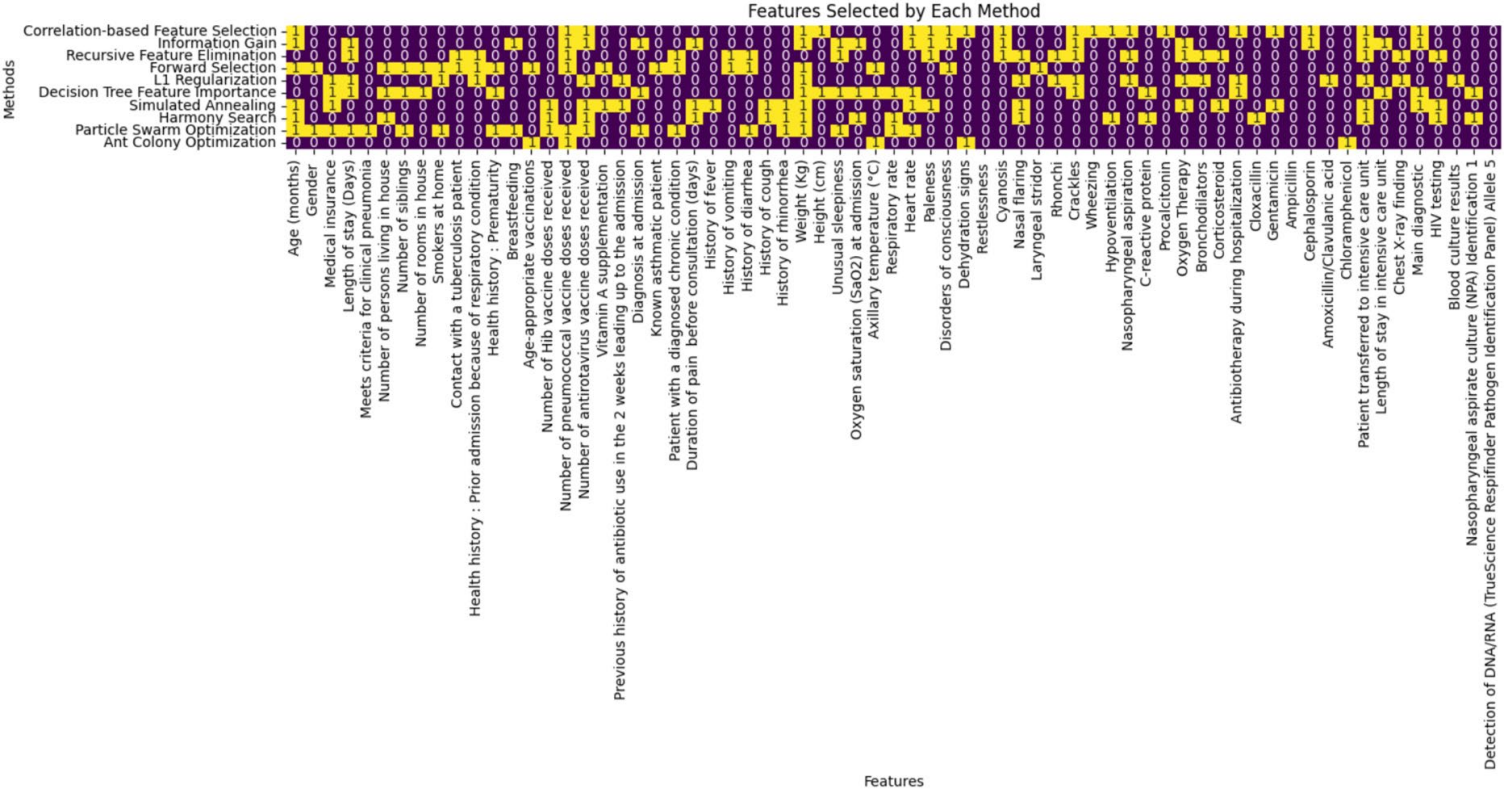
Features selected by each method.

Figure 2 depicts the 20 significant features that are selected by using Filter, Wrapper, Embedded, Physics-Inspired, and Nature-Inspired feature selection methods for further processing.

**Fig. 3:**
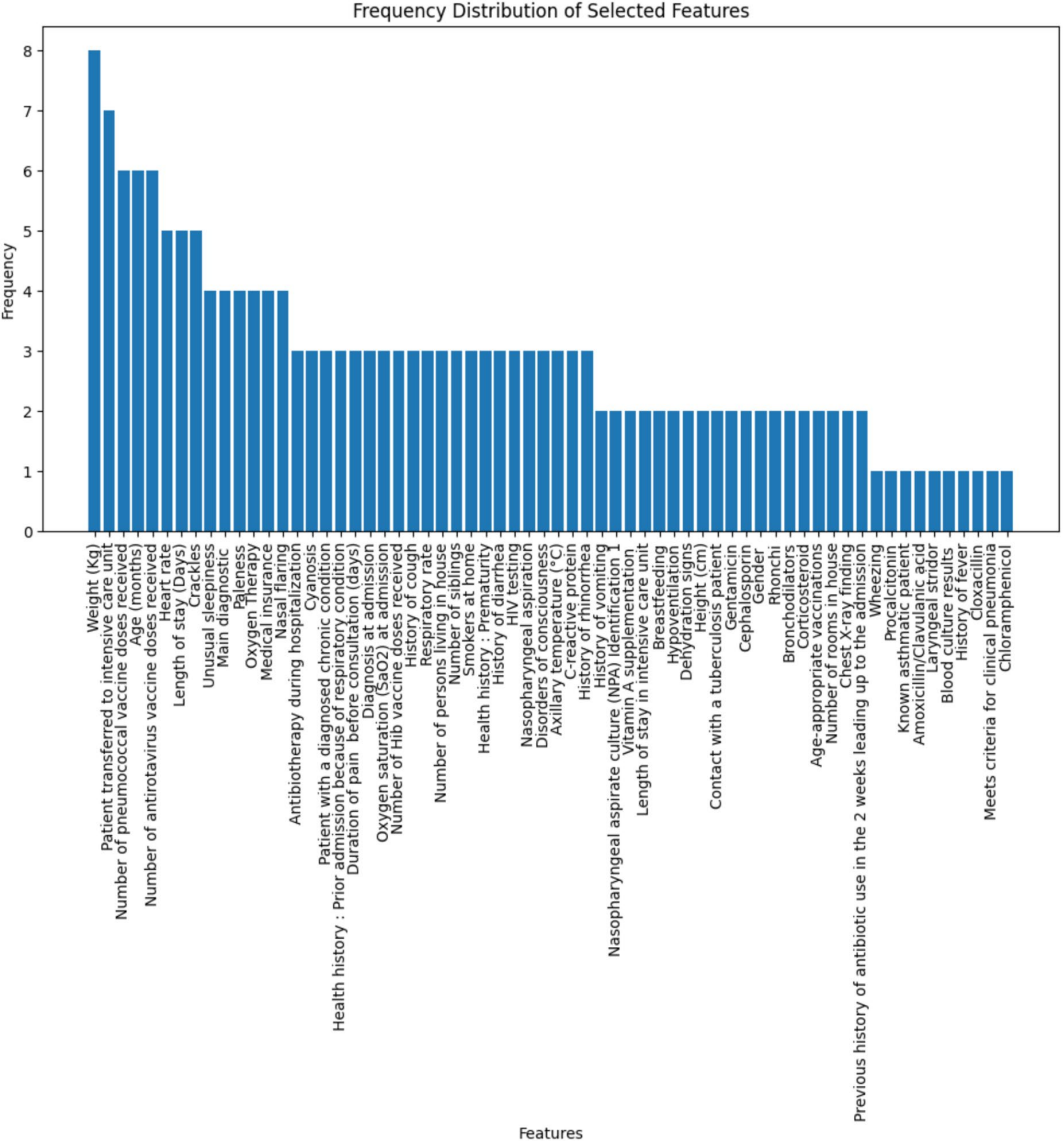
Frequency distribution of all selected features.

The frequency distribution of all the selected 20 features is plotted in Fig. 3, a single List is created by combining all the selected features, and the frequency of each feature is counted. Based on the count value, the top 15 features are selected and saved as a separate CSV file (top_15_features_with_target.csv) for further research.

Table 2 represents the data dictionary of the 15 most significant selected features that are designated as the final feature set.

**Table 2:** Data dictionary of the final-selected significant features.

S.No	Name of the feature	Type of the feature	Explanation
1	Patient transferred to intensive care unit	Binary	Used to denote whether a patient is transferred to ICU
2	Bronchodilators	Binary	Variable that represents whether Bronchodilators medications are used or not
3	Rhonchi	Binary	To represent the presence or absence of abnormal lung sounds Rhonchi
4	History of fever	Binary	To denote whether a patient got a fever before hospital admission or not
5	Unusual sleepiness	Binary	To represent the presence or absence of unusual sleepiness, drowsiness
6	Antibiotherapy during hospitalization	Binary	To store whether antibiotics were given to the patient or not
7	Wheezing	Binary	Used to store the presence or absence of wheezing
8	Oxygen therapy	Binary	To mention whether a patient received supplemental oxygen during hospitalization or not
9	Main diagnostic	Categorical	To represent the patient’s primary diagnosis at the time of admission
10	History of rhinorrhea	Binary	To store the presence or absence of rhinorrhea history
11	Chest X-ray finding	Categorical	To store the radiological observations made from a patient’s chest X-ray
12	Meets criteria for clinical pneumonia	Binary	To mention whether a patient was diagnosed with clinical pneumonia or not
13	Weight (Kg)	Numerical	Body weight of the patient (Kgs)
14	History of vomiting	Binary	To store whether a patient experienced vomiting before hospitalization or not
15	Vitamin A supplementation	Binary	To indicate whether a patient was Vitamin A supplemented or not

### Model training

This process is Like the heartbeat of machine learning, which will teach an algorithm how to make predictions or decisions based on the given inputs. This process will feed the model with training data, tweaking its internal parameters and refining its performance using various methods. This research uses 75% of the data for training and 25% for testing^32^. Standard ML algorithms that are listed in Table 3 are followed to build various models and 8 valuation metrics are considered for validating the models. As a technique for data augmentation, the Synthetic Minority Oversampling Technique (SMOTE) Medar et al.^53^ balances the imbalance variable “target variable: Clinical Progression"and helps for the better generalization of ML models across patient scenarios.

**Table 3:** Machine learning models considered for prediction.

S.No	Name of the algorithm	Explanation
1	Logistic regression	Binary classification through probabilistic estimation using a logistic function
2	Linear discriminant analysis	Method of dimensionality reduction and classification that maximizes class separability
3	Support vector machine (SVM)	A Supervised learning model that finds the optimal hyperplane for classification or regression
4	Decision trees	Tree-based model that makes a decision based on feature-based splits
5	Random forest	Ensemble learning by multiple decision trees to build a more accurate model and avoid overfitting
6	Gradient boosting machines	Iterative boosting algorithm that combines weak learners to make a strong predictive model
7	XGBoost	Gradient boosting framework optimized for performance and speed in machine learning applications
8	MultiLayer perceptron (MLP)	Deep learning-based artificial neural network learning complex patterns in data
9	Naive bayes	Probabilistic classifier using Bayes’ theorem with the independence assumption of simple and speed features
10	K-nearest neighbors	Non-parametric algorithm that classifies data based on the majority class of its nearest neighbors

To correct the issue of biased classes, the SMOTE projected only on the training set. This was done so as to ensure that no artificial samples would have affected the validation or test sets and thus data leakages would not occur. The use of SMOTE was not used on the whole dataset before splitting. This resourceful use prevents overfitting and the assessment of model performance. Prior to SMOTE, the dataset was heavily skewed (Cured: 94.50%, Dead: 3.74%, and Left against medical advice: 1.75%), which caused a high apparent accuracy as these models had a high proportion of the prediction of the majority class, and the minority classes had poor recall. The training set became a balanced one (33.3% per class) after SMOTE which made accuracy a significant point. The Top models improved as Random Forest: 95.52%, XGBoost: 94.52%, and Gradient Boosting: 92.03%.

### Evaluation of the models

The predictive quality of the proposed solution was evaluated by a set of ten machine learning models built on a balanced set of SMOTE datasets (75% training and 25% validation). It included not only high-level classification performance but also clinical reliability, using a combination of multiple metrics such as precision, accuracy, recall, F1-score, specificity, ROC-AUC, and log loss. Fitness (the probability of a model to appear at the top of the defined spectrum) was determined based on a weighting formula to obtain the best three of the models, which was later incorporated through a Soft Voting Ensemble to create a more stable model with less risk of pediatric clinical outcome misclassification prediction.

**Fig. 4:**
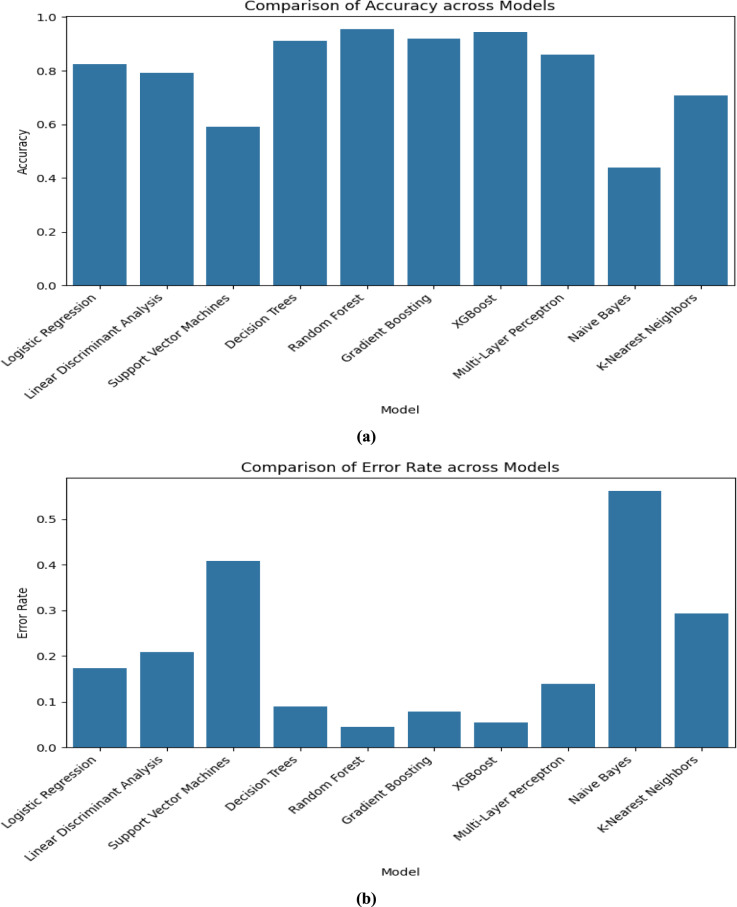
Bar chart comparing multiple models on (**a**) Accuracy and (**b**) Error Rate respectively.

**Fig. 5:**
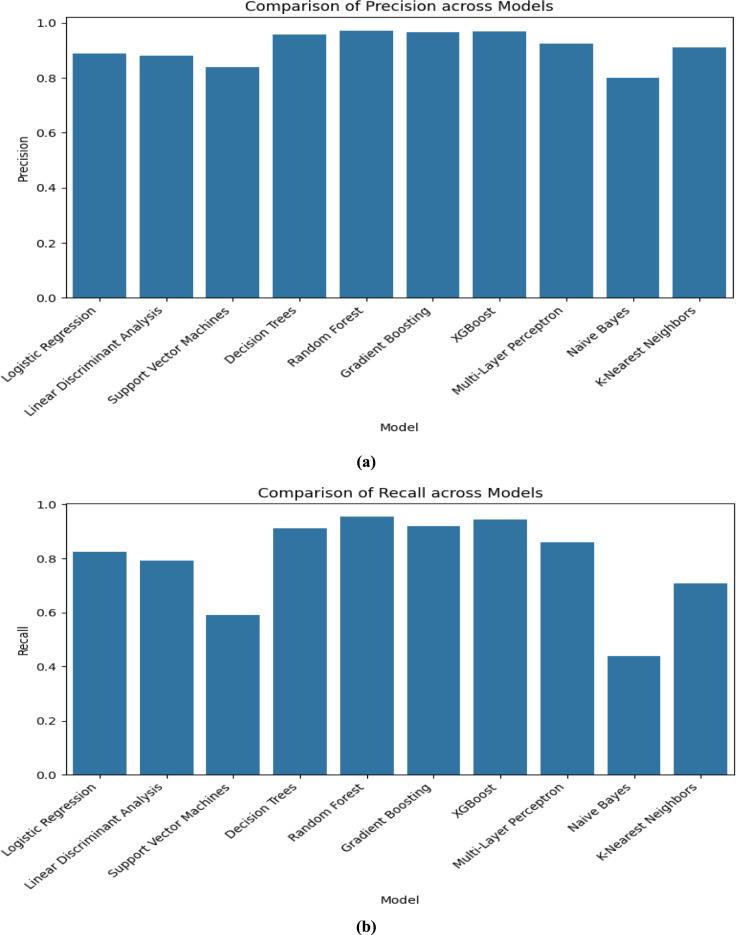
Bar chart comparing multiple models on (**a**) Precision and (**b**) Sensitivity respectively.

**Fig. 6:**
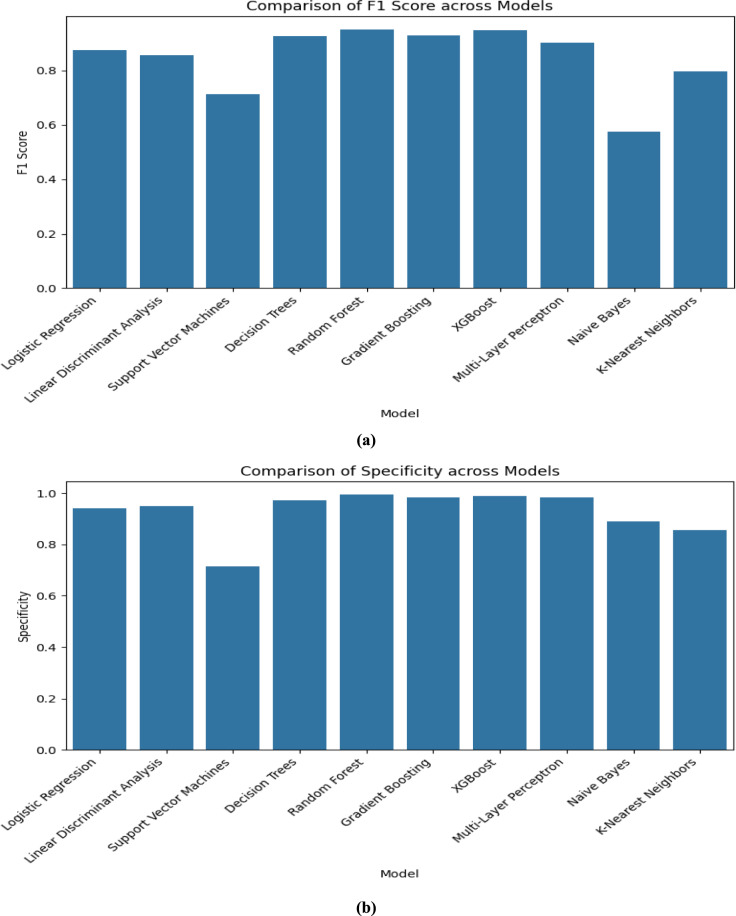
Bar chart comparing multiple models on (**a**) F1 Score and (**b**) Specificity respectively.

**Fig. 7:**
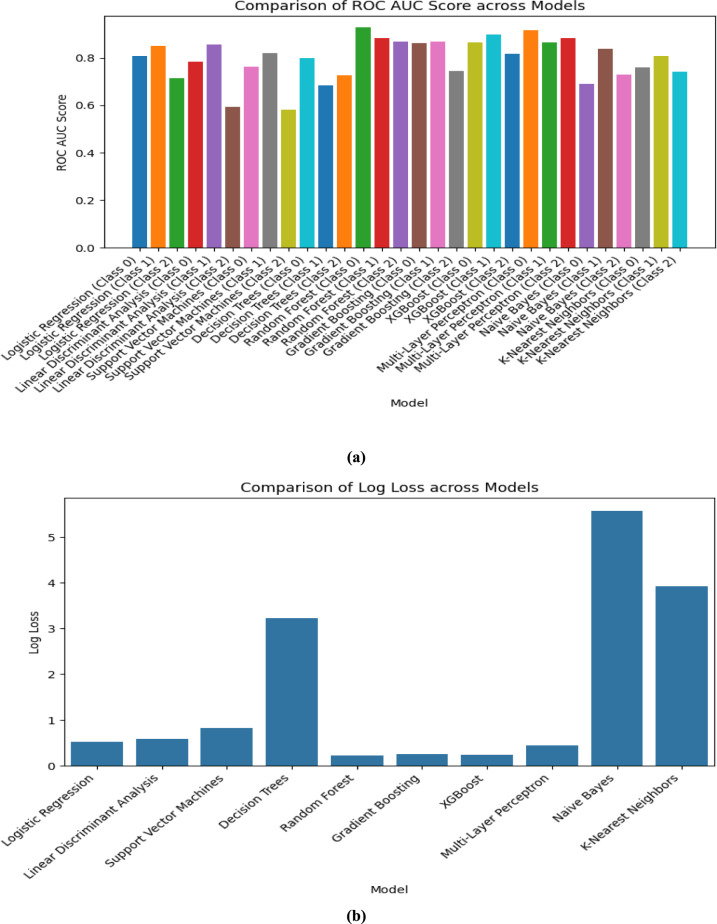
Bar chart comparing multiple models on ROC curve (color-coded) and (**b**) Log Loss respectively.

The performance of all the models that are listed in Table 3 is valuated and compared by using the visualization technique “Bar Graph” in this section by using the following Figs. 4, 5, 6 and 7. As we used “Bar Graph”, the value of all the performance metrics are given in the unit of “Percentage” (%).

In Fig. 4, the accuracy (Proportion of Correctly classified instances out of the total instances) and error rate (Proportion of Incorrectly classified instances out of the total instances) of all the considered models are compared by using a bar chart.

When we look at Fig. 4, we can observe that the Random Forest (95.52%), XGBoost (94.52%), and Gradient Boosting (92.03%) algorithms, attain high accuracy which indicates their strong predictive performance. The lowest accuracy is in Naïve Bayes model (43.78%) which means the algorithm is not fit for our research. The lowest error rate is observed in Random Forest (4.47%) and higher error rates in Naïve Bayes (56.21%) and SVM (40.79%).

In the following Fig. 5, the precision (Accuracy of positive predictions) is high for Random Forest (95.52%), XGBoost (94.52%), and Gradient Boosting (92.03%) which means these models made the fewest false positive predictions and Support Vector Machine (71.42%) predicted the high number of false positives.

At the same time, in the case of Recall/Sensitivity, Random Forest (95.52%), XGBoost (94.53%), and Gradient Boosting (92.04%) effectively identified positive instances compared with other models.

When we observe Fig. 6, the best F1-Scores were obtained by Random Forest (95.11%), XGBoost (94.71%), and Gradient Boosting (92.83%) which indicates a good balance between sensitivity and precision, and with the lowest F1 score (57.45%), Naïve Bayes performance was poor in classification. In the point of specificity Random Forest (99.47%) effectively classified negative instances.

ROC AUC (Receiver Operating Characteristics—Area Under Curve) Scores are presented in a Color-coded bar chart in Fig. 7 which shows Random Forest, XGBoost, and Gradient Boosting have excellent discriminatory ability than other models.

Log Loss denotes the uncertainty of a model’s probability predictions. In our work, Random Forest (0.22) and XGBoost (0.24) had the lowest log loss value which means they produced the most confident and accurate probability estimation.

When we analyze the above figures, for the best balance of accuracy, precision, recall, and log loss, Random Forest and XGBoost are the top choices, we can include Gradient Boosting also for complex datasets.

**Fig. 8:**
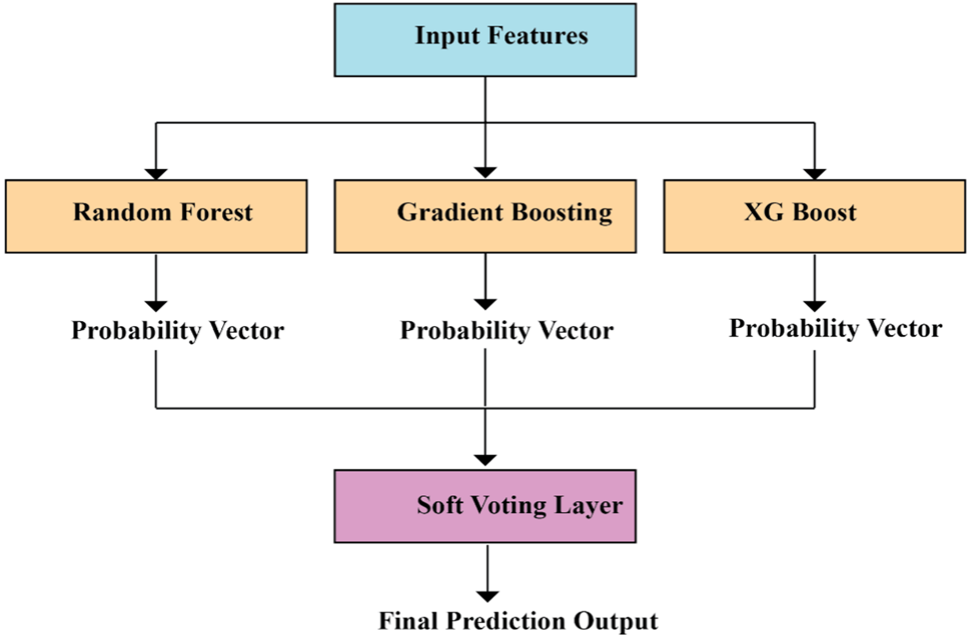
Soft voting ensemble architecture.

This research proposes a new step-by-step algorithm named “Algorithm for Optimized ML Model Selection and Ensemble Prediction” which is given in Fig. 8. The further flow of the research is just by following the algorithm that is given in Table 4.

**Table 4:**
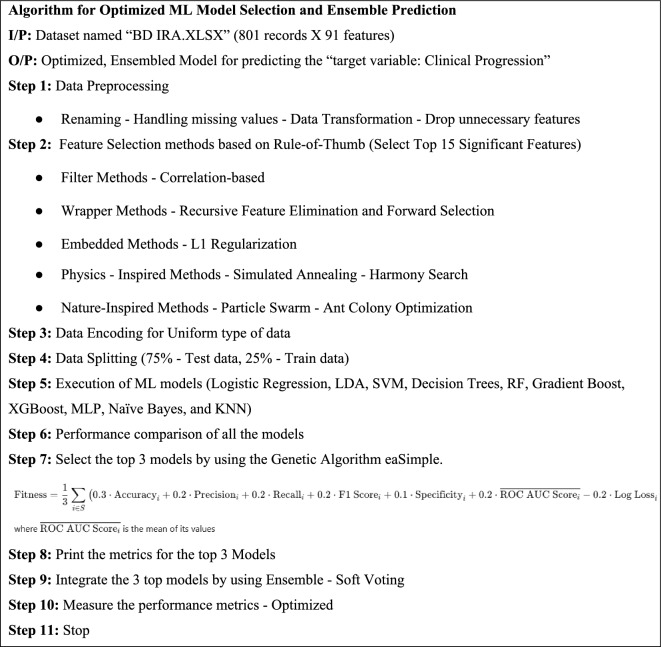
Algorithm for optimized ML model selection and ensemble prediction.

### Selecting the top 3 models using genetic algorithm

The Machine Learning models of this work used the selected significant features as input and predicted the output variable “target variable: Clinical progression”. Their performance level is measured and compared in Figs. 4, 5, 6 and 7. Selecting the optimal combination of 3 models from a potentially large pool of models is a combinatorial optimization problem. A genetic algorithm is a powerful, optimized, robust, and evolutionary method for exploring a large, complex search space to select the best performance model based on weighted fitness scores.

The Genetic Algorithm (GA) has been applied with the DEAP Python library (eaSimple), with the only purpose being to tell which of the three machine learning models performed best over all. The population size of the GA was 50 and it means that 5000 model combinations were examined during one generation. The algorithm was executed with 100 generations and this gave adequate iterations to cover the search space. Crossover between parent solutions to produce offspring was set to 0.5 and a mutation that was allowed was set to 0.2. Tournament selection was applied in order to make the selection diverse and to have individuals performing well. These parameters were selected as guided by the common practice in evolutionary optimization to achieve a good balance between convergence rate and exploration ability.

Eight metrics used in the fitness function were accuracy, precision, recall, F1 score, specificity, AUC, log loss, and error rate. The fitness score was formed as a weighted sum with all metrics being equally weighted after being normalized, and by exception log loss and error rate are subtracted as those metrics are inverses.

Fitness Score = w1⋅Accuracy + w2⋅Precision + w3⋅Recall + w4⋅F1 Score + w5⋅Specificity + w6⋅AUC − w7⋅Log Loss − w8⋅Error Rate.

Random Forest, XGBoost, and Gradient Boosting algorithms were chosen based on the fitness scores and, finally, predictive smooth voting was carried out.

The average weighted fitness is calculated by using the above formula. eaSimple (Evolutionary Algorithm Simple) is a simple, straightforward genetic algorithm approach that follows the evolutionary computation framework and includes selection, crossover, and mutation.

algorithms.eaSimple(population, toolbox, cxpb=crossover_prob, mutpb=mutation_prob,ngen=num_generations, stats=stats, halloffame=hof, verbose=True)

By using the above method, Random Forest, Gradient Boosting, and XGBoost are selected as the top 3 models. Their performance measures are given in the following Table 5.

**Table 5:**
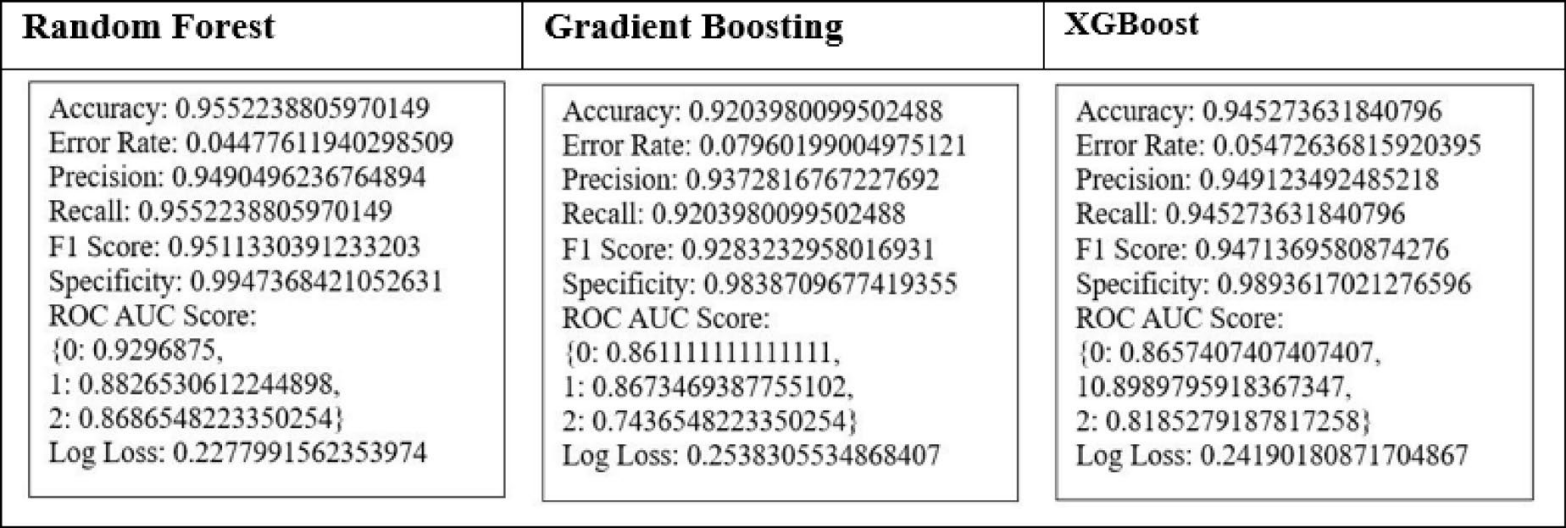
Performance evaluation measures for the top 3 models.

Ensembling is a technique to train the base models to solve a single problem and combine them to comprehend multiple models. This combined approach improves the combined model’s accuracy, robustness, and overall output.

Key features of Ensemble modeling:

- Reduced Variance and Improved Stability
- Better Generalization and Reliable Predictions
- Robustness to model failures
- Improvement on other metrics other than accuracy
- Handling Model Bias

The ensemble often provides a more robust, stable, and generalizable solution, which can be crucial in real-world applications where data can be noisy or vary over time.

As our research outcome is a classification variable, VotingClassifier is an apt ensemble learning type that can be used. VotingClassifier combines the predictions of multiple base models and produces the final output. There are two types of Voting Classifiers: Hard Voting and Soft Voting. We have used Soft Voting in our research as it aligns to our objective. Soft Voting follows the weighted average probability method, where each classifier outputs the probability for each class. The average of these probabilities is calculated and the class which has the highest probability will be selected as the final output. Precise pseudocode of the model selection process by using the GA is presented in Appendix A in order to facilitate understanding and reproduce the model selection process.

In our research, the top 3 models Random Forest, Gradient Boosting and XGB are combined by using the VotingClassifier—Soft Voting method to predict the output variable “target variable: Clinical progression” which is a categorical-classification output.

ensemble_model = VotingClassifier(estimators=[(‘rf’, model1), (‘gb’, model2), (‘xgb’, model3)], voting=‘soft’)

To further enhance the interpretability of the ensemble model, we analyzed the feature importance for each of the top three models (Random Forest, XGBoost, and Gradient Boosting). Random Forest showed that clinical features such as age, oxygen saturation levels, and respiratory rate were among the most influential in predicting patient outcomes. Similarly, XGBoost highlighted blood pressure and white blood cell count as critical for predicting mortality. The ensemble model, combining predictions from these models, demonstrated that age and oxygen saturation consistently contributed the most across all individual models.

**Fig. 9:**
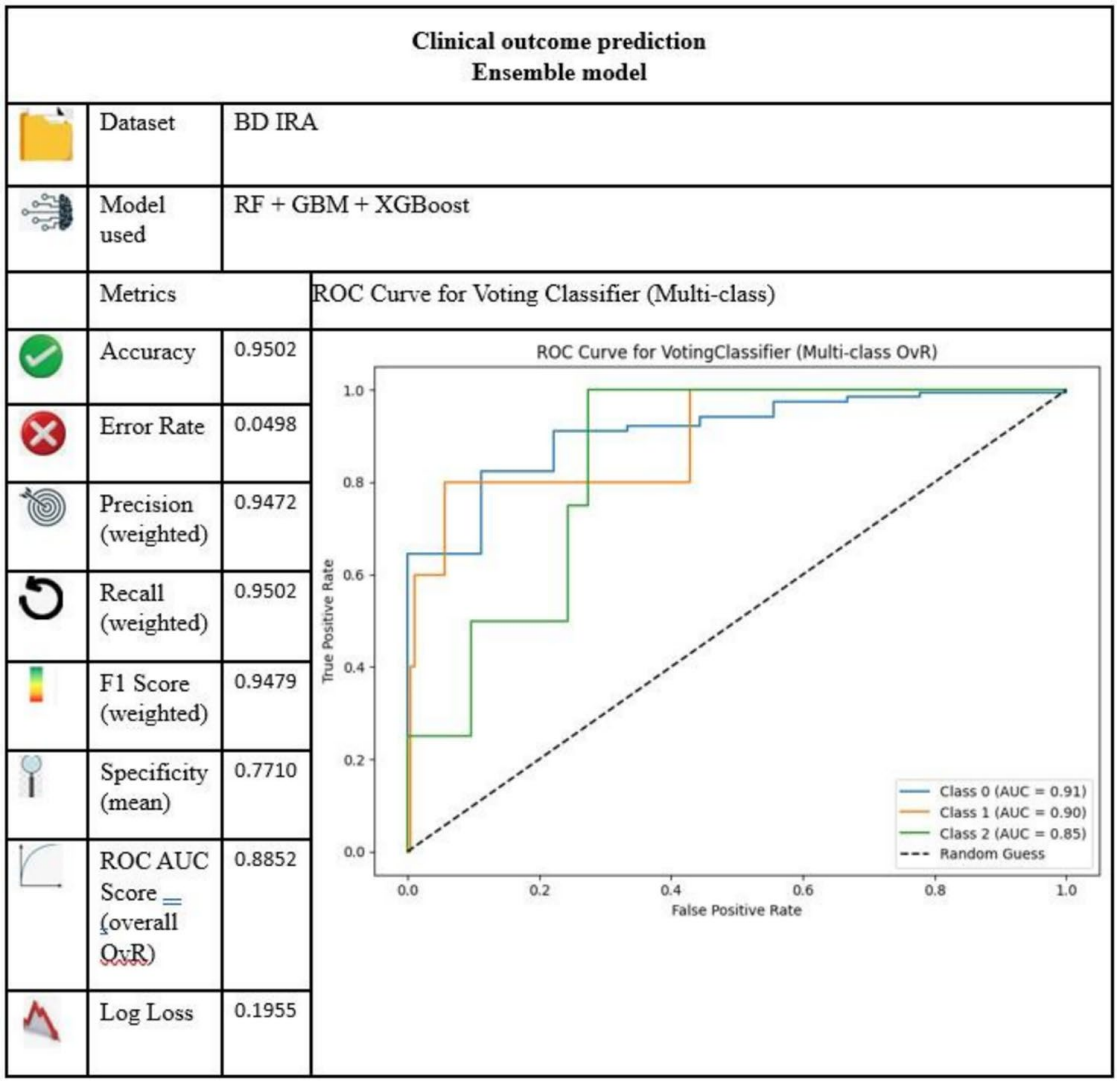
Performance measure of the proposed ensemble model.

Figure 9 gives the performance measures and ROC curve of the proposed model. The table shows that the ensembled model is optimized than the existing models in terms of specificity and Log Loss.

**Fig. 10:**
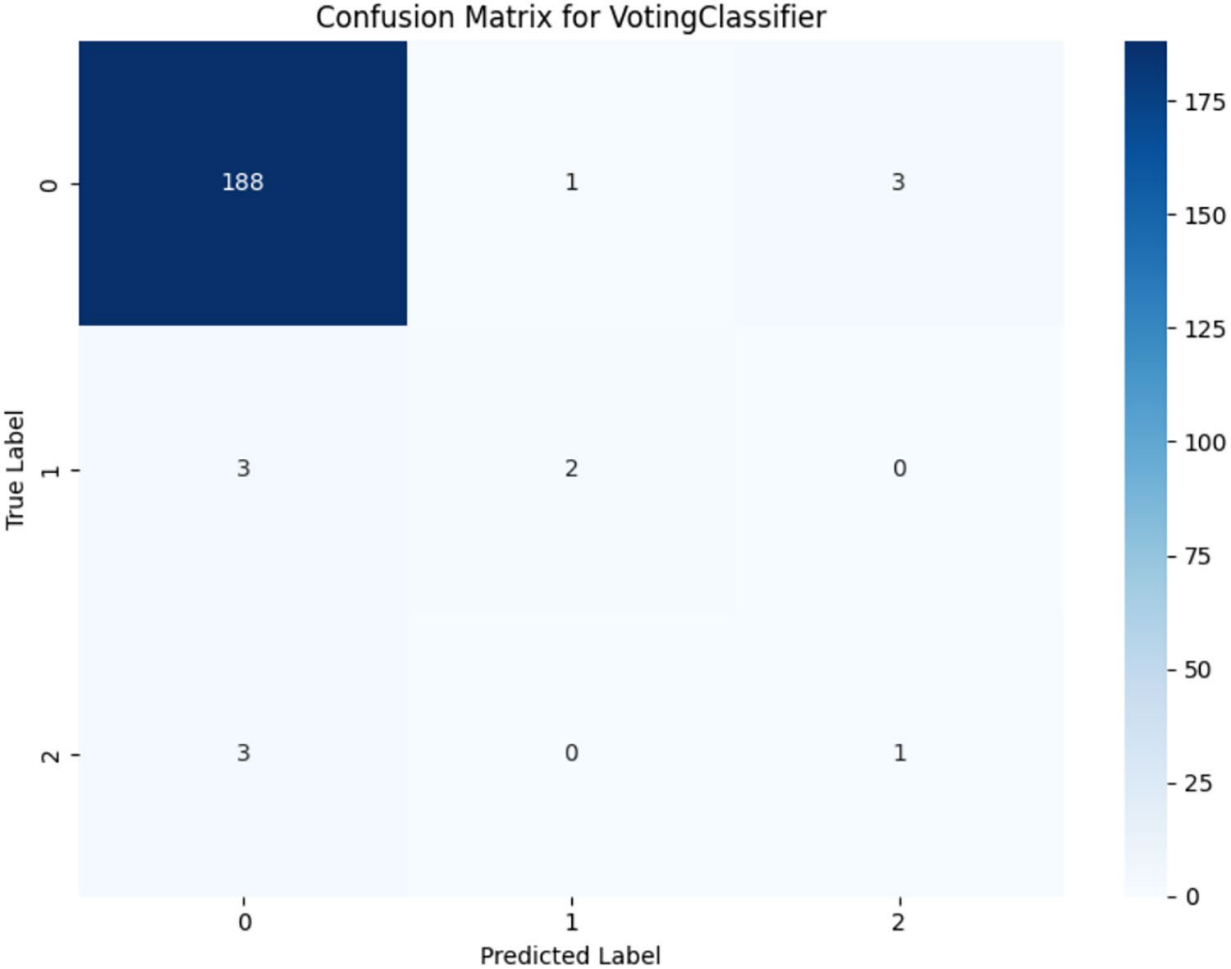
A confusion matrix for voting classifier.

With a confusion matrix of the proposed ensemble model as Fig. 10, it is clearly depicted by both the correct and the incorrect predictions on the three classes (Cured, Death, Left against medical advice). To emphasize on the performance to correctly identify the critical class (the Death class) the class-wise metrics (the Accuracy, Precision, Recall, F1-score and Specificity per class) are provided in Table 6.

**Table 6:** Class-wise metrics table.

Class-wise metrics					
Class 0		**Class 1**		**Class 2**	
Accuracy	0.9502	**Accuracy**	0.9801	**Accuracy**	0.9701
Precision	0.9691	**Precision**	0.6667	**Precision**	0.2500
Recall	0.9792	**Recall**	0.4000	**Recall**	0.2500
F1 Score	0.9741	**F1 Score**	0.5000	**F1 Score**	0.2500
Specificity	0.3333	**Specificity**	0.9949	**Specificity**	0.9848

The ensemble model’s consistently strong performance across all metrics suggests that it offers not only statistical improvement but also clinical relevance in predicting pediatric respiratory outcomes. Of course, the proposed, new model performance is not degraded in terms of other evaluation metrics. So, our proposed model is more effective than the previous research works. The direct clinical implications of these metrics are that high recall means that most pediatric patients associated with the risk of severe outcomes will be identified, high precision will prevent false alarms or prevent unnecessary intervention, and the AUC value in combination with low log loss means sufficient estimation of the probability.

Collectively, they indicate that the ensemble model is clinically useful with regard to the practical application of pediatric respiratory decision support.

To validate the proposed model, a paired samples t-test was carried out on evaluation metrics. A significance level of 0.05 was used to determine whether differences observed before and after SMOTE application were statistically meaningful. The findings indicated that the Synthetic Minority Over-sampling Technique (SMOTE) exerted a considerable positive influence on various metrics, including Accuracy, Error Rate, Precision, Recall, and Specificity.

### Limitations of the study

Despite the promising performance of the proposed model, several challenges remain when considering its deployment in clinical environments. Integration with electronic health records (EHRs) would require standardization of data formats, interoperability with existing clinical systems, and real-time processing capabilities. Furthermore, handling sensitive patient data involves strict adherence to privacy regulations such as HIPAA and GDPR, necessitating secure data pipelines and access control mechanisms.

The dataset comprised 801 pediatric cases from a single Moroccan tertiary hospital, collected over a one-year period. Although rich in clinical and etiological detail, its limited geographic and temporal scope restricts generalizability. The dataset also exhibits class imbalance, particularly in the “Death” and “Left against medical advice” categories. Another key consideration is the generalizability of the model across diverse hospital settings and populations, which may require domain adaptation or further validation with multi-institutional datasets. The dataset used, although clinically relevant, is relatively small and imbalanced, which may limit the generalizability of the findings to other populations or hospital settings. While SMOTE was employed to address class imbalance, the synthetic sampling may introduce noise or overfitting, despite ensemble techniques. Additionally, the model was evaluated on a single dataset without external validation, which may affect its robustness across varied clinical scenarios. Addressing these challenges is essential for translating the proposed system into a practical, scalable clinical decision support tool.Consequently, further studies using multi-center or multi-region datasets, coupled with external validation, would be required to confirm the generalizability and clinical utility of the approach.

## Conclusion

In the research, we proposed an optimized ML model for forecasting the class of target variable “target variable: Clinical progression” by using the other features of the collected dataset. This will be useful to the medical professionals to proceed with their further healthcare activities effectively. We reduced the dimensionality of the data and selected the impactful features. Standard ML models are executed with 75% testing and 25% training data and the top 3 models are selected by using genetic algorithms based on 8 evaluation metrics. The Ensemble approach is used to combine the selected top models, and it has been proven that the proposed experimentation using the BD IRA dataset yielded the optimal performance measures.

Future work should incorporate larger, multi-institutional datasets like sound, image, or any kind of multimedia medical records and include real-time validation to improve generalizability and clinical applicability. Also, we can integrate explainable AI (XAI) techniques such as SHAP (SHapley Additive exPlanations) and LIME (Local Interpretable Model-agnostic Explanations) to enhance the interpretability and clinical trustworthiness of the model’s predictions.

## Supplementary Information

Supplementary Information.

## Data Availability

The dataset used in this study is publicly available and can be accessed at: https://data.mendeley.com/datasets/2d9dvnycjw/1.

## Code availability

The code will be made publicly available after the work is accepted.
