# Supplementary material for: Clinical outcome prediction in pediatric respiratory infections using hybrid feature selection and a genetic algorithm-optimized machine learning
Source: Sci Rep. 2025 Oct 21;15:36542. doi: 10.1038/s41598-025-18258-6 (PMC12540805; doi:10.1038/s41598-025-18258-6)
Supplement: Supplementary file 1 — Supplementary Information. [file 41598_2025_18258_MOESM1_ESM.pdf]

APPENDIX A

Pseudocode of the model selection process by using the GA

**Input**	**:**	Set of candidate ML models
**Output**	**:**	3 best models of the ensemble
**Step 1**	**:**	Start with population (3-model combination),. n=50 random
**Step 2**	**:**	do generation = 1 to 100:<br>Measure fitness of individuals by means of:<br>Fitness = w1*Accuracy + w2*Precision + w3*Recall<br>+ w4*F1 + w5*Specificity + w6*AUC<br>- w7*LogLoss - w8*ErrorRate<br>Choose parents by means of the Tournament Selection<br>Use crossover (probability = 0.5) to make the offspring<br>To sustain diversity, use mutation (probability = 0.2)<br>Replenishment of old population with new population
**Step 3**	**:**	Return the best 3 models that achieve the highest fitness
